# Investigating Cerebello-Frontal Circuits Associated with Emotional Prosody: A Double-Blind tDCS and fNIRS study

**DOI:** 10.1007/s12311-024-01741-7

**Published:** 2024-09-14

**Authors:** Francesco Panico, Sharon Mara Luciano, Alessia Salzillo, Laura Sagliano, Luigi Trojano

**Affiliations:** University of Campania “Luigi Vanvitelli”, Viale Ellittico 31, 81100 Caserta, Italy

**Keywords:** Emotional prosody, Non-invasive brain stimulation, Cerebellum, Prefrontal cortex, Functional connections

## Abstract

The emotional and cognitive cerebellum has been explored by several studies in the past years. Recent evidence suggested the possible contribution of the cerebellum in processing emotional prosody, namely the ability to comprehend the emotional content of a given vocal utterance, likely mediated by anatomical and functional cerebello-prefrontal connections. In the present study, the involvement of a functional cerebello-prefrontal network in recognising emotional prosody was assessed by combining non-invasive anodal transcranial direct current stimulation (tDCS) over the right or the left cerebellum and functional Near Infrared Spectroscopy of the prefrontal cortex, in a double-blind within-subject experimental design on healthy participants. The results showed that right and, to a less extent, left cerebellar tDCS (as compared to sham stimulation) reduced neural activation in the prefrontal cortex while accuracy and reaction times at the vocal recognition task remained unchanged. These findings highlight functional properties of the cerebello-frontal connections and the psychophysiological effects of cerebellar brain stimulation, with possible clinical applications in psychiatric and neurological conditions.

**Supplementary Information:**

The online version contains supplementary material available at. 10.1007/s12311-024-01741-7.

## Introduction

In the last decades, a plethora of studies demonstrated the role of the cerebellum in cognition and affect [1]. Classical studies showed that posterior cerebellar lesions can produce deficits in executive functions, visual spatial processing, linguistic skills, and regulation of affect, thus giving rise to the clinical picture termed the Cerebellar Cognitive Affective Syndrome [2, 3]. In healthy individuals, early functional magnetic resonance imaging (fMRI) studies demonstrated that the posterior cerebellum is active while categorising visual images eliciting primary emotions [4]. In the field of non-invasive brain stimulation, Ferrucci et al. [5] showed that cerebellar transcranial Direct Current Stimulation (tDCS) was able to selectively enhance processing of negative facial expressions; moreover, a consistent body of evidence demonstrated that Transcranial Magnetic Stimulation (TMS) over the cerebellum can affect mood regulation, emotion discrimination, and the processing of social information [6–9]. On these bases, several reviews and consensus papers outlined that the cerebellum is involved in many cognitive domains, particularly in executive control, linguistic processing, experience and regulation of emotional states, and social cognition [10–12]. Indeed, it has been proposed that the cerebellum participates also in the processes associated with mentalising, theory of mind, and body reading (for a recent consenus paper see [13]).

Very recently, a few studies have focused on the role of the cerebellum in processing emotional prosody [14–17], which is defined as the changes in the intonation, loudness and tempo of speech conveying the emotional content of an utterance, independently from its linguistic content [18]. Although an extended fronto-temporal network, mainly in the right hemisphere, is thought to be involved in the processing of emotional prosody [18, 19], several studies pointed to an important contribution of the cerebellum in this process. For instance, in a fMRI study, Pitchon & Kell [20] found that the production of emotional prosody (as compared to production of neutral speech) increased neural response within the inferior frontal gyrus, the thalamus and globus pallidus, substantia nigra, the superior temporal sulcus and, crucially, the cerebellum. The activation in this network was observed bilaterally but to a much larger extent in the right hemisphere. As for recognition of emotional prosody, neuroimaging studies described the involvement of a network encompassing the temporal and frontal cortices, the basal ganglia and the cerebellum [17, 21, 22], but with inconsistent evidence about laterality of cerebellar involvement. Indeed, a left [21], right [22] or bilateral [17] cerebellar involvement has been reported by different studies. In clinical populations, Thomasson et al. [15] reported an impairment in recognising emotional prosody conveying surprise or fear in patients with chronic cerebellar ischaemic stroke (as compared to healthy controls). In this last study, lesion-symptom mapping revealed that these impairments were mainly associated with lesions in right posterior cerebellar lobules [15]. An impaired performance in emotional prosody recognition was confirmed by further studies from the same group on patients with cerebellar stroke or Parkinson’s disease, with a larger involvement of the right cerebellum [14, 23].

The contribution of the cerebellum to processing emotional prosody, as well as to other cognitive and affective functions, seems to be crucially related to its structural and functional connections with the rest of the brain. Indeed, distinct output spots within the cerebellum project to, and receive feedback from, several cortical areas, constituting closed-loop cerebello-cerebral interactions [1, 24, 25]. Recently, Krienen and Buckner [26] identified four topographically distinct cerebello-cerebral circuits involving the frontal cortex in humans. These circuits seem to represent an anatomical and functional correlate supporting a role of the cerebellum in cognition and emotional processing [26]. Importantly, many recent studies suggested that an alteration in the functioning of cerebello-frontal circuits could be associated with abnormal emotion processing in psychopathological [27, 28] and neuropsychological conditions [14, 15, 29, 30]. Using resting-state fMRI, Brady et al. [27] identified a network centred on cerebello-frontal structures associated with negative symptoms in patients with schizophrenia; moreover, repetitive modulation using TMS over the cerebellum was able to restore within-network connectivity and ameliorate severity of negative symptoms [27].

These findings call for the development of valid and reliable methods of brain recording and stimulation allowing to target and possibly modulate neural activity within the cerebellum and the associated networks involved in cognitive and emotional processing, as resting-state fMRI and TMS are not always widely available in both research and clinical contexts [31, 32]. Moreover, such techniques pose some constraints (e.g. regarding the posture to be kept during the experiment) that limit their possible applications. For this reason, it would be meaningful to develop more easily applicable tools to investigate and modulate cerebello-frontal connections. Functional Near Infrared Spectroscopy (fNIRS) has been recently proposed as a sensitive method for assessing haemodynamic response associated with neural activation, by measuring changes in oxygenated (O2HB) and deoxygenated (HHB) haemoglobin. This technique allows gauging neuronal activity in real-time with reasonable spatial and good temporal resolution and adequate sensitivity, is robust to various artifacts, and yet is much cheaper than fMRI, easier to implement, and can be used in clinical contexts, combined with any kind of task [33]. Importantly, fNIRS can be used simultaneously with non-invasive stimulation to measure the changes induced in brain activity without notable interference. For this reason, several studies have recently combined fNIRS with tDCS, which in turn represents a technique allowing to modulate brain activity non-invasively [31, 32]. As compared to TMS, tDCS has the advantage of being easily portable and more friendly-to-use, thus allowing a wider range of applications in research and rehabilitative contexts. Although some previous studies have shown the potential of using both fNIRS and tDCS during experimental tasks and in resting state experimental designs [for a review see 31], the possibility to use these two techniques to target the activity of the cerebello-prefrontal network effectively has not been explored.

The aim of the present study is twofold. First, we wanted to investigate the possible involvement of the cerebellum during recognition of emotional prosody, also addressing the issue of lateralisation of cerebellar involvement. Second, we aimed to explore the possibility to assess and modulate the functional circuits involving the cerebellum and the prefrontal cortex (PFC) by using easily replicable methods (namely fNIRS and tDCS). To fulfil these purposes, a group of healthy participants completed an auditory task requiring recognition of the emotional content conveyed by meaningless vocal utterances, in a within-subject double-blind study involving three online stimulation conditions targeting the cerebellum (anodal stimulation of the right cerebellum, anodal stimulation of the left cerebellum, sham stimulation). Before and after each stimulation session, the neural activation in the PFC was recorded in resting state to measure possible changes due to stimulation of the cerebello-frontal network. Based on the literature cited above on emotional prosody [15, 19, 20] and on the cerebello-frontal networks [31], we hypothesised that non-invasive tDCS of the cerebellum, particularly of the right cerebellum (as compared to sham), could modulate emotion recognition from voice and affect brain activity in the PFC. These findings would support the involvement of the cerebellum in emotional prosody and would provide evidence of the possibility to modulate cerebello-prefrontal functions by the combined used of fNIRS and tDCS, thus opening to relevant applications in cognitive, affective, and rehabilitative neurosciences.

## Materials and Methods

### Participants and Experimental Design

In a within-subject, double-blind, online tDCS design, the participants completed three experimental sessions, one week apart; during each session the participants underwent tDCS, while performing the voice emotion recognition task. Before and after the stimulation session, prefrontal brain activity was recorded by means of a wearable fNIRS device.

Twenty right-handed university students (age range= 18-29; average age= 22.75, SD= 3.09; 10 female) voluntarily participated in the study. An a priori power analysis conducted with G*Power 3 [34], set the minimum total sample size at 14 participants to conduct repeated measures ANOVAs with two within-subject factors (emotion: 6 levels; stimulation: 3 levels; see thereafter) and detect an effect size of 0.25, with a power of 0.95, and an alpha of 0.05. Participants had normal or corrected-to-normal vision, had no history of neurological or psychiatric disease, and were naïve to the purposes of the study. At enrolment, all the participants completed an interview confirming their eligibility and providing their written informed consent to take part in the stimulation study. The procedure was in agreement with 1975 Helsinki Declaration and was approved by the Ethic Committee of the Department of Psychology (n. 21/2023).

### Vocal Emotion Recognition Task

The vocal emotion recognition task was implemented in PsyToolkit software [35]. Participants were exposed to audio recordings of human voice, selected from an Italian emotional speech database (EMOVO Corpus [36]), in which professional actors produced brief meaningless sentences using a different emotional intonation. Each audio recording lasted 2.7-3 s. We selected four meaningless sentences, each pronounced by four different actors (two male and two female) for each of six emotions (happiness, rage, sadness, surprise, disgust, and neutral). Thus, the task included a total of 96 stimuli (4 sentences x 4 voices x 6 emotions) pseudorandomised in two blocks of 48 stimuli. Audio recordings were presented binaurally using stereo headphones; audio was fixed at the same intensity (65%) for all participants. Each block was separated by a 1-minute break. Sentences in each block were presented to participants in a random order. A training phase (n=16 trials) preceded the experimental task to allow participants to familiarise with the instructions and to check they could hear the stimuli correctly; the stimuli used in the training phase were not included in the experimental task. Participants had to respond on a numeric keyboard by pressing one key number (1 to 6) corresponding to the emotion they felt was expressed by the voice (maximum time allowed= 5000 ms). Inter-stimulus interval was set at 500 ms. Response keys for emotions were counterbalanced, and the starting position was kept constant in a centrally positioned key (i.e. key number 0). Response could be provided by participants only at the end of each audio recording. No feedback on accuracy was provided. All participants responded using their right hand. The entire task lasted about 18 minutes. The same task was used in the three experimental sessions.

### transcranial Direct Current Stimulation (tDCS)

Stimulation started three minutes before the beginning of the vocal emotion recognition task and lasted through it (online protocol mode). A constant current of 2.0 mA intensity was delivered by a battery-driven stimulator (BrainSTIM, EMS Medical, Italy) using a pair of 5x5 surface saline-soaked sponge electrodes (area= 25 cm2). The anode was placed over the right or left cerebellar lobe (with the centre of the electrode aligned 3 cm lateral to the inion), while the cathodal electrode was placed over the ipsilateral buccinator muscle [37]. This monocephalic montage was chosen to avoid possible confounding effects due to modulation of a further brain area. Sham stimulation was performed in the same way as active stimulation, but the stimulator was turned off after 30s, so to ensure that participants felt the same itching sensation at the beginning of tDCS as in the other experimental sessions and were thus blinded on the stimulation they were receiving [38]. In the sham stimulation condition, the “anodal” electrode was placed over the right cerebellum in half participants and over the left cerebellum in the other half. Overall stimulation duration time was set at 21 minutes (including 30s of ramp in and rump out phases) and complied with safety guidelines on tDCS use [39]. An investigator not involved in recruitment, experimental protocol, and analysis set the stimulation condition according to a predetermined randomisation sequence.

### functional Near Infrared Spectroscopy (fNIRS)

**Fig. 1:**
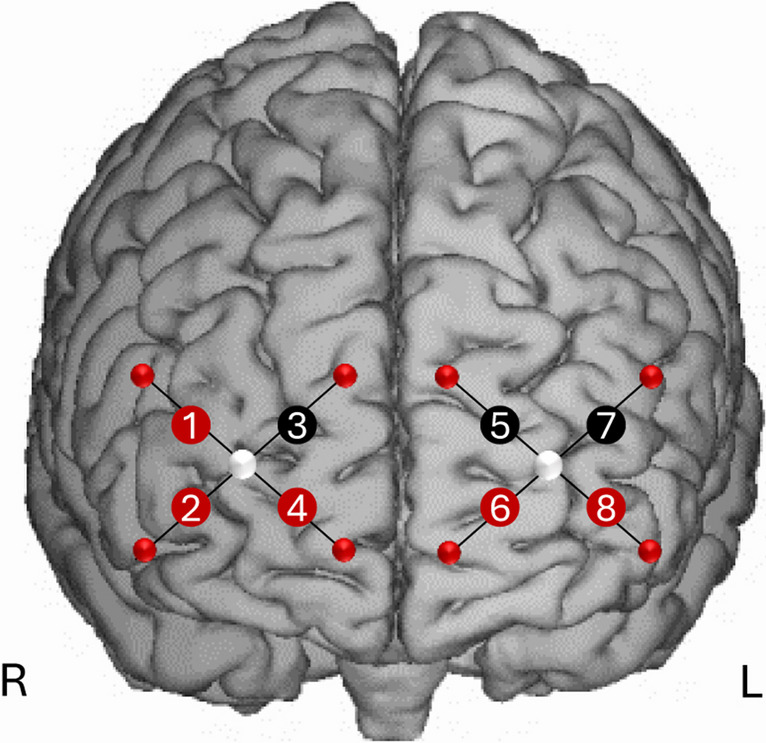
Location of the optodes on participants’ forehead from channel 1 to channel 8. The headband included two receivers (in the centre) and eight transmitters (in the periphery). Channels highlighted in red (coloured figure in the online version of the paper) showed significant reduction in O2HB levels following real as compared to sham stimulation. R and L indicate the right and left hemispheres

We assessed brain activity in the PFC by means of a wearable fNIRS device for three minutes in resting state before and after the stimulation session [31]. A 2 × 4-channel continuous wave fNIRS system (OctaMon, Artinis Medical Systems, The Netherlands) was employed to record levels in O2Hb and HHb over the bilateral PFC (Fig. 1). The device measures the variations in light attenuation at two wavelengths (758 and 840 nm). The O2Hb and HHb concentration levels (expressed in ΔμM), obtained using the modified Beer–Lambert law, were displayed in real time. Data were acquired using the OxySoft software (OxySoft, Artinis Medical Systems, The Netherlands) at a frequency of 10 Hz. The differential pathlength factor (DPF) was selected individually for each participant according to the age [40]. Eight LEDs bundles (four for each hemisphere) were utilised to carry out the light to the left and the right PFC, whereas two photodiodes (one for each hemisphere) with proprietary ambient light protection were used to collect the light emerging from the same cortical areas. The detector–illuminator distance was set at 35 mm. This allowed to have eight recording channels (right hemisphere: Ch 1-4; left hemisphere: Ch 5-8). The bundles were assembled into a probe holder that kept fixed the position of the optodes. The probe holder was placed over the head to include the underlying PFC with the two photodiodes receivers aligned on Fp1 and Fp2 locations (see [41]) according to the international 10–20 system for the electroencephalography electrode placement. The probe holder provided a stable contact with the scalp for all the optodes. However, optical contact was monitored continuously during the protocol.

### Visual Analogue Scale for Mood (VAS)

To control for possible effects of simulation on the subjective evaluation of affective and physiological state of participants, at the beginning and at the end of each experimental session the participants filled in a questionnaire using visual analogue scales (VAS) implemented in Psytoolkit software [35]. Questions targeted the following domains: happiness, sadness, calm, tense, tiredness, and sleepiness (e.g.: ‘how happy do you feel right now?’). Each VAS consisted of a horizontal line, 100 mm in length, anchored at each end by word descriptors. The participants marked the point on the line they felt best represented how they perceived their current state. The VAS score was calculated by measuring in millimetres the distance from the left side of the line to the point that the participants marked. The VAS score ranged 0(minimum)-100(maximum).

### Data Analysis

#### Accuracy and Reaction Times (RTs)

The sum of Errors for each participant and the mean RTs for correct responses at the vocal emotion recognition task were computed. For RTs, datapoint below or above 2 standard deviations from the mean were removed and treated as missing data. Two repeated measures Analyses of Variance (ANOVAs) were performed on mean total Errors and mean RTs with the variables Emotion (happiness vs rage vs sadness vs surprise vs disgust vs neutral) and Stimulation (anodal r-Cb vs anodal l-Cb vs sham) as within-subject factors.

#### fNIRS Preprocessing and Analysis

Signal quality as well as the absence of movement artifacts were visually inspected [42]. fNIRS data were then pre-processed in OxySoft using a Moving Gaussian filter at 1Hz [42, 43]. O2Hb and HHb measures were then averaged across the first minute of resting state recording performed before and after each experimental session using OxySoft. An index of variation in neural activation was calculated by subtracting O2Hb (and HHb) values in the post as compared to the pre (Post-Pre), with positive values indicating and increase in neural activation and negative values indicating a reduction in neural activation following stimulation.

Separate ANOVAs on O2Hb and HHb indices of activation derived from each channel (from Ch 1 to Ch 8) were performed with Stimulation (anodal r-Cb vs anodal l-Cb vs sham) as within-subject factor; Bonferroni-corrected post-hoc tests were adopted. To summarise data, we also collapsed data recorded in the regions of interests (ROIs) for each hemisphere and conducted secondary repeated-measure ANOVAs on O2Hb (and HHb) indices of activation with the factor ROI (left vs right hemisphere) and Stimulation (anodal r-Cb vs anodal l-Cb vs sham) as within-subject factors.

#### VAS

To evaluate changes in mood and psychophysiological state possibly associated with stimulation, a delta variation (Post-Pre) on the VAS scores was calculated for happiness, sadness, calm, tiredness, and sleepiness as dependent variables. Separate repeated-measures ANOVAs were performed on the delta change in the VAS scores with Stimulation (anodal r-Cb vs anodal l-Cb vs sham) as within-subject factor.

All analyses were performed in JASP software (version 0.18.3.0). The level of significance was set at 0.05 and post hoc comparisons were adjusted using Bonferroni correction.

## Results

### Accuracy and RTs

Repeated-measure ANOVA on errors at the vocal recognition task showed a significant main effect of Emotion [F(5,90)=30.7, *p*<.001, η^2^=.51; Table 1], but no significant main effect of Stimulation [F(2,36)=.82, *p*=.45, η^2^=.00] or EmotionXStimulation interaction [F(10,180)=.84, *p*=.59; η^2^=.01]. Post-hoc comparisons showed that the number of errors in recognition of surprise (Table 1) was significantly higher than the number of errors for sadness (*p*<.001), rage (*p*<.001), happiness (*p*=.002) and neutral stimuli (*p*<.001); no difference was observed with respect to disgust (*p*=.71). Errors in recognising disgust were higher as compared to rage (*p*<.001), and sadness (*p*<.001) and neutral stimuli (*p*<.001), while no difference was found with respect to happiness (*p*=.66). Moreover, errors in recognizing happiness were higher as compared to rage (*p*<.001), and sadness (*p*=.01) and neutral stimuli (*p*<.001). The number of errors for rage and sadness did not differ (*p*=.19).

**Table 1:** Mean errors (and SE) during the Vocal Emotion Recognition Task as a function of Stimulation and Emotion

	sham		r-Cb		l-Cb		Total	
	Mean	SE	Mean	SE	Mean	SE	Mean	SE
Sadness	3.53	.39	4.16	.51	4.37	.51	4.02	.41
Disgust	7	.48	7.32	.62	7.32	.58	7.21	.41
Happiness	5.84	.53	5.90	.44	6.32	.48	6.02	.41
Neutral	3.26	.61	3.74	.58	3.53	.55	3.51	.41
Rage	2.42	.52	2.58	.44	2.58	.44	2.53	.41
Surprise	8.63	.53	8.79	.48	7.74	.46	8.39	.41
Total	5.11	.21	5.41	.19	5.31	.25		

The ANOVA on RTs revealed a significant main effect of Emotion [F(5,55)=3.87, *p*=.004; η^2^=.04], but no main effect of Stimulation [F(2,22)=.07, *p*=.93; η^2^=.004] or interaction between Emotion and Stimulation [F(10,110)=.07, *p*=1.00; η^2^=.002]. Post hoc comparisons showed significantly shorter RTs in recognising sadness (Table 2) as compared to surprise (*p*=.006), rage (*p*=.045), and disgust (*p*=.02). All the remaining comparisons were not significant (all p>.05).

**Table 2:** RTs for correct answers (and SE) at the Vocal Emotion Recognition Task as a function of Stimulation and Emotion

	sham		r-Cb		l-Cb		Total	
	Mean	SE	Mean	SE	Mean	SE	Mean	SE
Sadness	870.98	73.85	870.43	73.16	884.05	69.85	875.16	54.69
Disgust	1004.11	108.55	986.21	100.75	1005.05	130.19	998.45	47.50
Happiness	992.94	52.41	947.80	66.71	1001.08	94.07	980.61	54.98
Neutral	930.32	76.29	909.81	99.96	974.87	89.18	938.33	67.64
Rage	986.78	71.09	978.65	86.60	1002.90	65.06	989.44	59.35
Surprise	1031.70	69.17	988.33	93.40	1022.99	90.87	1014.34	55.67
Total	969.47	66.13	946.87	73.25	981.82	81.47		

### Haemodynamic Response

**Fig. 2:**
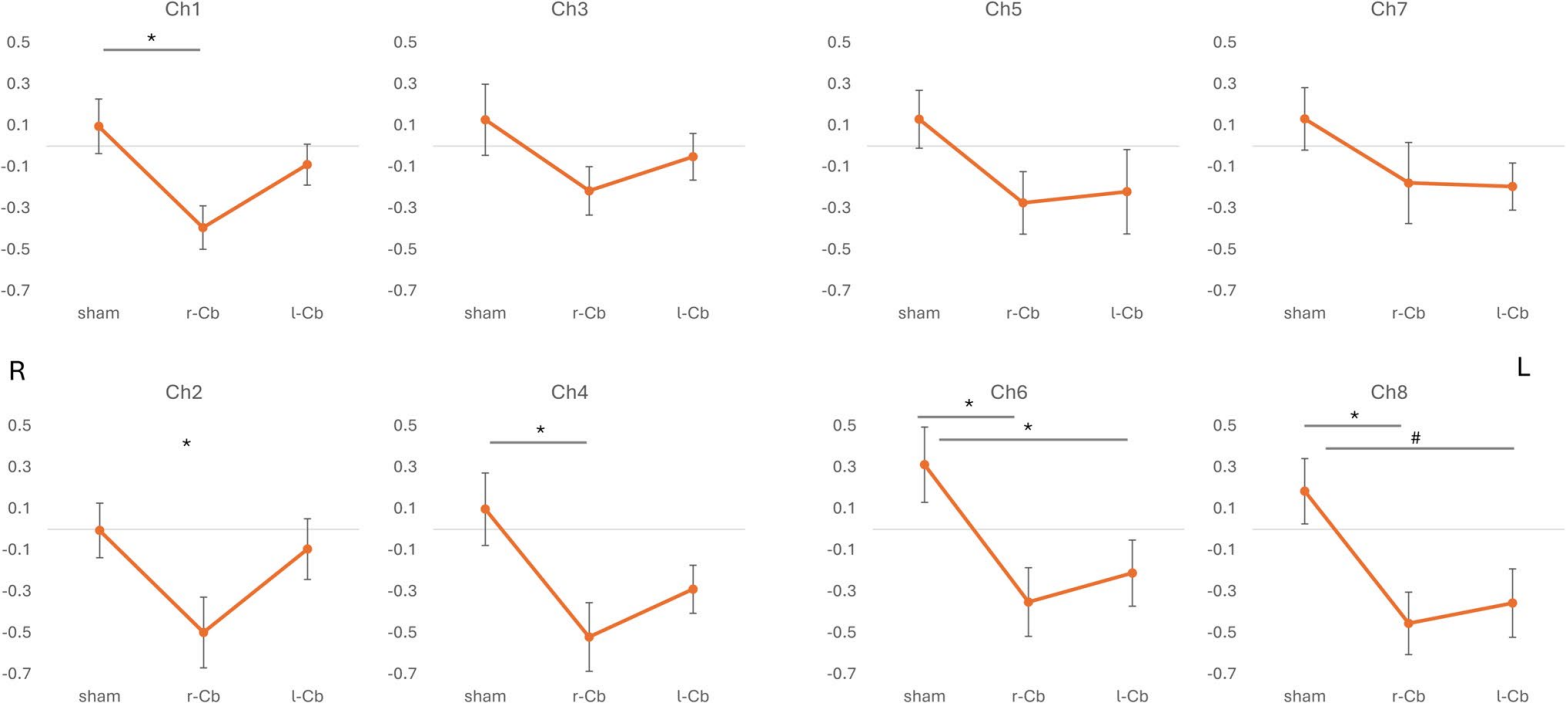
Results from ANOVAs on O2HB changes in the PFC (from channel 1 to 8) following cerebellar stimulation (sham, active right cerebellar stimulation, and active left cerebellar stimulation). R and L indicate location of channels in the right and left hemispheres respectively; * indicates significant at *p*<.05; # indicates a tendency to significance. Coloured figure in the online version of the paper

fNIRS data from two participants were removed from the analyses due to bad quality signal. Mean values of O2HB and results from the ANOVAs on each channel are reported in Table 3. A significant main effect of Stimulation was observed for Ch1, Ch2, Ch4, Ch6, and Ch8 (Fig. 1). For all these channels, post hoc comparisons showed a reduction of O2HB levels in the PFC following r-Cb stimulation as compared to sham stimulation (Ch1 *p*=.008; Ch2 *p*=.055; Ch4 *p*=.012; Ch6 *p*=.009, and Ch8 *p*=.023; Figure 2). No significant difference (all p>.05) was observed between r-Cb and l-Cb or between l-Cb and sham stimulation in all channels but Ch6, where O2HB levels were significantly lower after l-Cb stimulation as compared to sham (*p*= .048; Figure 2), whereas O2HB levels did not differ after l-Cb and r-Cb stimulation (p>.05). Finally, slightly lower O2HB levels after l-Cb stimulation as compared to sham stimulation were observed for Ch8 (*p*=.065; comparison between r-Cb and l-Cb stimulation not significant, p>.05; Figure 2).

**Table 3:** O2HB changes for each channel in the PFC following cerebellar stimulation and summary of ANOVA

	sham		r-Cb		l-Cb		Main effect (stim)	
	Mean	SE	Mean	SE	Mean	SE	F (2, 32)	*p*-value
Ch1	.096	.132	-.394	.105	-.089	.099	5.397	**.010**
Ch2	-.006	.132	-.499	.171	-.096	.147	3.493	**.042**
Ch3	.128	.172	-.216	.117	-.051	.113	1.969	.156
Ch4	.097	.175	-.521	.166	-.290	.116	4.957	**.013**
Ch5	.130	.140	-.274	.151	-.220	.204	1.591	.219
Ch6	.312	.182	-.352	.166	-.212	.160	5.766	**.007**
Ch7	.132	.151	-.178	.196	-.195	.114	1.325	.280
Ch8	.184	.158	-.455	.151	-.357	.165	4.722	**.016**

The ANOVA on O2HB contrasting data collapsed from the ROIs for the left and right hemisphere showed a significant main effect of Stimulation [F(2,32)=5.59, *p*=.008; η^2^=.21], with a reduction of O2HB levels in the PFC following r-Cb stimulation (M=-.36, SE=.12) as compared to sham stimulation (M=.13, SE=.12; *p*=.007); the comparisons between r-Cb and l-Cb stimulation (M=-.19, SE=.12), and between l-Cb stimulation and sham stimulation were not significant (*p*=.78 and *p*=.12 respectively). No other significant effect was observed [main effect of ROIs: F(1,16)=.47, *p*=.51; η^2^=.001; interaction between Stimulation and ROIs: F(2,32)=1.35, *p*=.27; η^2^=.01].

Mean values of HHB derived from each channel and results from ANOVAs are reported in Supplementary Table 1 and depicted in Supplementary Figure 1. The channel-by-channel analysis showed small variations in HHB values across the experimental conditions, and ANOVAs showed no significant differences in HHB levels as a function of the stimulation delivered (all p>.05). The ANOVA contrasting data gathered from the ROIs of the left and of the right hemisphere showed slightly higher HHB levels in the PFC following r-Cb stimulation as compared to sham stimulation, a pattern complementing that observed for O2HB, and higher levels of HHB in the left as compared to the right hemisphere (see Supplementary File).

### Mood and Psychophysiological State

Mean variations at VAS scales following the stimulation sessions are reported in Supplementary Table 2. ANOVAs on delta variation of VAS scores showed no significant effect related to stimulation for happiness [F(2,38)=.87, *p*=.42; η^2^=.04], sadness [F(2,38)=1.26, *p*=.29; η^2^=.06], calm [F(2,38)=.41, *p*=.66; η^2^=.02], tense [F(2,38)=1.54, *p*=.23; η^2^=.07], tiredness [F(2,38)=.97, *p*=.39; η^2^=.05], or sleepiness [F(2,38)=.05, *p*=.95; η^2^=.003].

## Discussion

Based on recent studies on the role of the cerebellum in cognitive and affective functions [1, 3, 12, 13, 44], here we aimed at evaluating the possibility to assess and modulate the activity in cerebellar-prefrontal circuits during processing of emotional prosody by means of non-invasive, easily applicable, reliable, and relatively inexpensive psychophysiological techniques, such as fNIRS and tDCS. We expected that tDCS could affect the behavioural performance in terms of errors and RTs during a vocal recognition task, and modulate activation in the prefrontal region, as measured by haemodynamic levels. We controlled for possible confounding effects due to changes in subjective evaluation of affective or physiological state by means of participants’ self-ratings on their own current state.

### Effect of Cerebellar Stimulation on Emotional Prosody Recognition and Mood

Behavioural measures showed that participants made a higher number of errors when recognising emotional stimuli conveying surprise, disgust, and happiness as compared to processing of neutral utterances. Moreover, the participants were slower in recognizing surprise, disgust and rage as compared to sadness. However, the type of cerebellar stimulation delivered in the three sessions had no significant effect on number of errors or RTs; subjective evaluation of affective and physiological states too did not change as a function of stimulation condition.

The lack of effect of stimulation condition on the vocal recognition task does not fit our hypotheses and findings from some previous clinical [14, 15] and fMRI [20] studies. Moreover, a previous experimental study by Ferrucci et al. [5] reported that both anodal and cathodal cerebellar tDCS can affect recognition of emotion from *facial* expressions in healthy participants, with shorter reaction times for negative facial expressions as compared to positive and neutral facial expressions (no effect on errors was observed in the study). Some differences in the experimental designs could be relevant to explain the lack of effect in the present study. In our experiment we used a multiple-choice response modality, whereas other studies employed continuous visuo-analogue scales to rate emotional valence in order to obtain a more sensitive measurement for each emotion [15, 45]. Moreover, Ferrucci et al. [5] used an offline stimulation protocol, with the task being performed 35 minutes following stimulation, whereas here the vocal recognition task was executed concurrently with tDCS stimulation. Future studies are warranted to ascertain whether our null behavioural findings are related to a weak sensitivity of the response modality or to the stimulation protocol, or to the relatively lower contribution of the cerebellum in recognising emotional prosody [14, 15, 20], differently from other aspects of emotional processing [5, 10, 44]. It is worth noting that several neurostimulation studies provided evidence of the possibility to interfere with processing of affective and social information by applying TMS over the cerebellum [6–9]. It would be interesting to directly compare effectiveness of tDCS and TMS in modulating the emotional cerebellum.

### Modulation of Cerebellar-Frontal Circuits by Non-invasive Stimulation

Although we did not observe a significant effect on behavioural measures, the present paper provides the first demonstration of the possibility to use tDCS over the cerebellum for modulating neural activity in the PFC, i.e. outside the primary motor cortex which has been investigated in previous studies (see below). Indeed, and importantly, analyses of the haemodynamic response showed that stimulation of the right, and, to a less extent, of the left cerebellum reduced activation in the PFC as compared to sham stimulation. These results will be discussed in the context of anatomical and functional studies on cerebellar-cortical connections and of studies addressing the properties and the outcomes of the stimulation techniques. The discussion of fNIRS data will focus on O2Hb, which seems to provide better contrast and higher variations as compared to HHb [46].

First, previous studies using viral tracing techniques have identified multiple segregated cerebello-frontal circuits in non-human primates [47, 48]. In humans, fMRI connectivity studies identified four topographically distinct cerebello-frontal circuits, three of which including the PFC and one involving the motor cortex [26]. However, these highly relevant data did not provide information on directionality of connections, given the intrinsic correlational nature of the technique. Our results are well in keeping with the evidence of cerebro-cerebellar connections described by previous studies [26, 47–49], but also demonstrated the possibility for the cerebellar stimulation to modulate neural activation in cortical regions within the PFC. These results are in keeping with recent resting-state fMRI and TMS studies reporting a modulation in cerebello-frontal circuits associated with affective processes [27, 28], but have been obtained by means of easily available, portable, and simpler tools as compared to fMRI and TMS.

Second, the channel-to-channel analyses showed that both right and left cerebellar stimulation reduced activation in the ipsilateral PFC whereas only right cerebellar stimulation could determine a reduction in activation of the contralateral PFC. This is compatible with the functional organisation of cerebellar-frontal circuits proposed by Krienen and Buckner [26], who maintained that although the connections between the cerebellum and the frontal cortex are preferentially crossed, the 20-30% of them terminate ipsilaterally. Thus, it is possible that the cerebellar stimulation delivered in our experimental design, covering an entire cerebellar lobule, may have influenced contralateral and ipsilateral cerebellar-frontal connections broadly. It would be worth investigating whether advanced high-definition stimulation protocols may be suitable to target these connections more precisely. Moreover, the right cerebellar stimulation induced changes in a greater number of channels, also in the contralateral PFC, compared to left cerebellar stimulation. The secondary analyses contrasting cumulated data from the channels of the right and of the left hemispheres confirmed the higher efficacy of the right cerebellar stimulation in modulating brain activity in the PFC. These observations may be interpreted by considering that tDCS has been shown to exert a state-dependant modulatory effect, affecting brain networks activated by ongoing behavioural tasks. Indeed, tDCS does not induce activity in resting neuronal networks, but can modulate neuronal activity, with dimension and direction of effects depending on the current physiological state of the target neural structures [32, 50, 51]. It is thus possible that the more robust effects following right cerebellar stimulation may be due to a functional involvement of a right-lateralised brain network activated by the emotional processing task which participants were engaged in, consistent with previous evidence [14, 15, 18, 19, 22, 23]. This speculation should be specifically tested by studies using behavioural tasks engaging different brain networks.

A third important issue deals with the polarity changes induced by the stimulation. Indeed, we applied anodal stimulation, which is thought to have excitatory effects, over the cerebellar lobules and obtained a reduction in the levels of activation in the PFC. This finding can be discussed in light of a very recent study by Shoaib et al. [24] who investigated the effect of anodal tDCS over the cerebellum on the activity of the primary motor cortex, as measured by fNIRS, using an experimental procedure very similar to the present one. In Shoaib et al.’s study, right anodal cerebellar stimulation induced a significant decrease in O2HB concentration in the contralateral (left) motor cortex and an increase in O2HB concentration in the ipsilateral (right) motor cortex, as compared to sham, indicating both inhibitory and excitatory effects depending on the considered hemisphere [24]. These polarity differences in the motor cortices of the two hemispheres were interpreted by the Authors with reference to a mechanism of inter-hemispheric inhibition, where the decrease in the left motor cortex activity was associated with a parallel increase of activity within the right motor cortex. Our results confirmed a reduced contralateral activity in the frontal cortex following right anodal cerebellar stimulation. However, they differ from those obtained by Shoaib et al. [24] as we observed an inhibitory effect on both the left and right PFC, suggesting a possible different functional organisation within these regions in terms of inter-hemispheric facilitatory and inhibitory processes. We might speculate that while a mechanism of inter-hemispheric inhibition is strongly represented within the motor cortex to subserve accurate execution of movements and motor learning [52], this process would not be represented within the PFC likewise. Indeed, although some cognitive and affective processes have been shown to be preferentially lateralised in the two hemispheres, a mechanisms of inter-hemispheric inhibition may be less central in the cerebello-prefrontal network [53, 54]. Future studies are needed to compare functional connectivity between the cerebellum and the motor and prefrontal cortex together with the relative peculiarities. It is also relevant to mention that other studies demonstrated either null, inhibitory or excitatory effects of cerebellar stimulation over the motor cortex depending on the stimulation parameters being used and the measures considered [55–57], also in line with evidence from the broader field of neurostimulation [58]. Moreover, factors such as anatomical differences in the scalp of individuals, and physiological and electrical activity differences among individuals could contribute to explain variability in the effects of non-invasive neuromodulation [59].

Considering previous anatomical studies, connections between the cerebellum and the cortex should be mediated by the dentate nucleus, the pons and the thalamus which represent relay stations between the cerebellum and the neocortex [26, 47, 48]. In terms of functional activation, the inhibitory effect of the cerebellum over the PFC could be explained hypothesising an increase in excitation of the Purkinje cells resulting in inhibitory output from deep cerebellar nuclei and a decrease in facilitation of thalamic cortical structures [24, 60]. In the present case, the widespread stimulation targeting an entire cerebellar lobe, makes difficult to trace the neuromodulatory mechanisms of cerebellar stimulation [61, 62]. Nonetheless, the present data pave the way for further investigation of this issue.

### Conclusions, Limitations, and Future Directions

The results from the present study demonstrated that anodal electrical stimulation of the cerebellum can reduce haemodynamic response in the PFC, providing evidence for specific cerebello-cortical functional connections involving the PFC. These results should be taken with caution, because of some study limitations. We did not monitor activity in the PFC during the whole stimulation time and during the emotional prosody task, so we could not obtain cues on the timing and dynamics of functional connections between the cerebellum and the PFC. As PFC recordings were not performed during task completion, no inference can be drawn about the involvement of this region in processing emotional prosody. The use of a block design with fNIRS recordings during the emotional prosody task and concurrent stimulation could help address this issue. Moreover, the spatial resolution of the tDCS and fNIRS devices did not allow to obtain stringent clues on the specific connectivity between the cerebellar lobules and the regions in the PFC. Concerning cerebellar stimulation, Rezaee and Dutta [63] have recently investigated the electric field distribution of two tDCS montages similar to the one used in the present experiment targeting the lateral cerebellum; the electric field strength of both montages affected the lobules Crus I/II, VIIb, VIII, and IX, which are thought to be relevantly involved in emotional processing [12]. With reference to the fNIRS data, our results from channel-to-channel analyses suggested that the ventro-medial and orbital prefrontal cortex showed the main functional changes following cerebellar stimulation. This finding is in line with evidence from the animal [28, 64] and human [65, 66] models demonstrating an involvement of the connections between the cerebellum and ventromedial and orbitofrontal areas in emotional and social processing. However, a higher number of channels and of sensitivity analyses are needed for obtaining higher spatial resolution and support this hypothesis, whereas the use of anodal and cathodal stimulation in the same experimental setting [5] will allow to explore polarity effects of cerebellar stimulation on downregulation of prefrontal activity. It will be also useful to ascertain whether different task paradigms can make evident possible behavioural effects of cerebellar stimulation on auditory emotion recognition, which we could not detect.

Aside from the above limitations, this study opens to possible relevant applications in cognitive, affective, and rehabilitative neurosciences. Indeed, as we provided evidence that cerebellar stimulation can affect prefrontal activity following just one session of stimulation, future studies involving repeated tDCS stimulation sessions could explore the possibility to modulate cognitive, emotional and social cognition impairments associated with cerebellar involvement as described in schizophrenia [27], autism spectrum disorder [28], Parkinson disease [30] and multiple sclerosis [67].

## Supplementary Information

Below is the link to the electronic supplementary material.

Supplementary file1 (DOCX 88 KB)

## Data Availability

Data will be provided upon reasonable request. Code availability: the script of the experimental task will be provided upon reasonable request

## Code availability

The script of the experimental task will be provided upon reasonable request

## Declarations

### Open practice statement

Data or materials for the experiments are available upon request, and none of the experiments was preregistered.
