# Supplementary material for: Investigating Cerebello-Frontal Circuits Associated with Emotional Prosody: A Double-Blind tDCS and fNIRS study
Source: Cerebellum. 2024 Sep 14;23(6):2397–407. doi: 10.1007/s12311-024-01741-7 (PMC11585498; doi:10.1007/s12311-024-01741-7)
Supplement: Supplementary file 1 — Supplementary file1 (DOCX 88 KB) [file 12311_2024_1741_MOESM1_ESM.docx]

**Supplementary Files**

Investigating cerebello-frontal circuits associated with emotional prosody: a double-blind tDCS and fNIRS study

Francesco Panico, Sharon Mara Luciano, Alessia Salzillo, Laura Sagliano, Luigi Trojano

*University of Campania “Luigi Vanvitelli”, Viale Ellittico 31, 81100 Caserta, Italy*

**Supplementary Table 1**. Levels of HHB for each channel in the PFC following cerebellar stimulation and summary of ANOVAs

|  | sham | | r-Cb | | l-Cb | | Main effect (group) | |
| --- | --- | --- | --- | --- | --- | --- | --- | --- |
|  | Mean | SE | Mean | SE | Mean | SE | F (2, 32) | p-value |
| Ch1 | .054 | .096 | .111 | .053 | .004 | .052 | .561 | .576 |
| Ch2 | -.025 | .046 | .074 | .072 | .104 | .057 | 1.354 | .273 |
| Ch3 | .059 | .069 | .087 | .080 | -.013 | .040 | .693 | .507 |
| Ch4 | -.083 | .050 | .088 | .073 | -.096 | .088 | 2.060 | .144 |
| Ch5 | .014 | .074 | .191 | .075 | .136 | .067 | 1.790 | .183 |
| Ch6 | -.021 | .059 | .223 | .083 | .072 | .073 | 2.372 | .109 |
| Ch7 | -.033 | .067 | .152 | .062 | .029 | .069 | 1.766 | .187 |
| Ch8 | .039 | .045 | .231 | .086 | .099 | .055 | 2.167 | .131 |

r-Cb= right cerebellar anodal stimulation; l-Cb= left cerebellar anodal stimulation

**Supplementary Table 2.** Mean variations at Visual Analogue Scales following stimulation **s**essions

|  | sham | | r-Cb | | l-Cb | |
| --- | --- | --- | --- | --- | --- | --- |
|  | Mean | SE | Mean | SE | Mean | SE |
| happiness | 3.70 | 2.33 | -0.25 | 2.28 | 2.10 | 3.17 |
| sadness | 2.75 | 3.72 | -6.15 | 4.34 | -0.65 | 4.49 |
| calm | 3.55 | 3.61 | 3.20 | 3.63 | 8.10 | 4.75 |
| tense | -4.20 | 2.70 | -9.60 | 4.85 | -16.60 | 5.59 |
| tiredness | 4.50 | 3.69 | -1.40 | 5.21 | -2.35 | 4.65 |
| sleepiness | 6.80 | 3.33 | 8.65 | 5.61 | 8.90 | 5.52 |

r-Cb= right cerebellar anodal stimulation; l-Cb= left cerebellar anodal stimulation


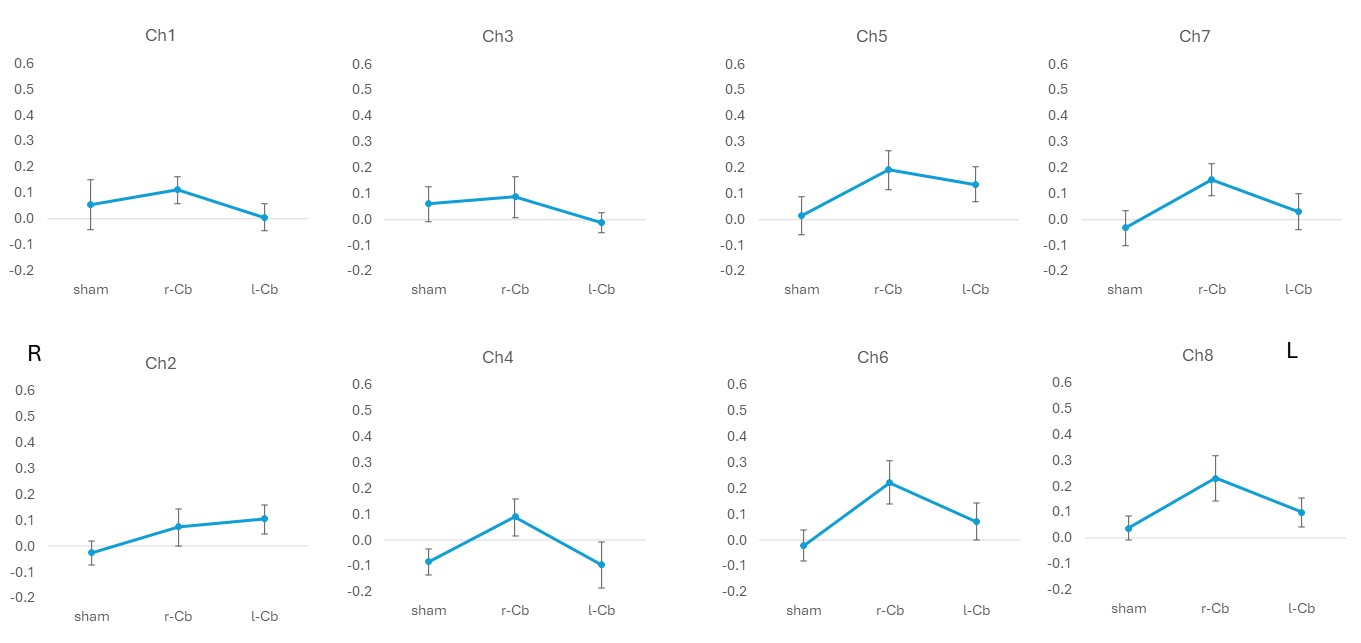


**Supplementary Figure 1**. Results from ANOVAs on HHB changes in the PFC (from channel 1 to 8) following cerebellar stimulation (sham, active right cerebellar stimulation, and active left cerebellar stimulation). R and L indicate location of channels in the right and left hemispheres respectively; no significant difference was found according to stimulation condition. Coloured figure in the online version of the paper.

**Results from the 3x2 repeated measures ANOVA on HHB**

A repeated measures ANOVAs on HHb indices of PFC activation with the factor ROI (left vs right hemisphere) and Stimulation (anodal r-Cb vs anodal l-Cb vs sham) as within subject factors was conducted. The results showed a significant main effect of Stimulation [F(2,32)=3.36, p=.047; η²=.11], with a tendency to an increase of HHB levels in the PFC following r-Cb stimulation (M=.18, SE=.05) as compared to sham stimulation (M=.001, SE=.05; p=.056); comparison between r-Cb and l-Cb stimulation (M=.04, SE=.05), and between l-Cb and sham stimulation were not significant (p=.19 and p=1 respectively). Moreover, a significant main effect of ROI was found [F(1,16)=5.52, p=.03; η²=.04], with increased levels of HHB in the left as compared to the right hemispheres (M=.12, SE=.03; M=.03, SE=.03; p=.03). No interaction between Stimulation and ROI was observed [F(2,32)=2.11, p=.14; η²=.03].
